# Molecular Diagnostic Yield of Exome Sequencing in Patients With Congenital Hydrocephalus: A Systematic Review and Meta-Analysis

**DOI:** 10.1001/jamanetworkopen.2023.43384

**Published:** 2023-11-22

**Authors:** Ana B. W. Greenberg, Neel H. Mehta, Garrett Allington, Sheng Chih Jin, Andrés Moreno-De-Luca, Kristopher T. Kahle

**Affiliations:** 1 Department of Neurosurgery, Massachusetts General Hospital, Boston; 2 Department of Pathology, Yale University School of Medicine, New Haven, Connecticut; 3 Department of Genetics, Washington University School of Medicine, St Louis, Missouri; 4 Department of Pediatrics, Washington University School of Medicine, St Louis, Missouri; 5 Department of Radiology, Neuroradiology Section, Kingston Health Sciences Centre, Queen’s University Faculty of Health Sciences, Kingston, Ontario, Canada; 6 Broad Institute of MIT and Harvard, Cambridge, Massachusetts; 7 Harvard Center for Hydrocephalus and Neurodevelopmental Disorders, Massachusetts General Hospital, Boston

## Abstract

**Importance:**

Exome sequencing (ES) has been established as the preferred first line of diagnostic testing for certain neurodevelopmental disorders, such as global developmental delay and autism spectrum disorder; however, current recommendations are not specific to or inclusive of congenital hydrocephalus (CH).

**Objective:**

To determine the diagnostic yield of ES in CH and whether ES should be considered as a first line diagnostic test for CH

**Data Sources:**

PubMed, Cochrane Library, and Google Scholar were used to identify studies published in English between January 1, 2010, and April 10, 2023. The following search terms were used to identify studies: *congenital hydrocephalus*, *ventriculomegaly*, *cerebral ventriculomegaly*, *primary ventriculomegaly*, *fetal ventriculomegaly*, *prenatal ventriculomegaly*, *molecular analysis*, *genetic cause*, *genetic etiology*, *genetic testing*, *exome sequencing*, *whole exome sequencing*, *genome sequencing*, *microarray*, *microarray analysis*, and *copy number variants*.

**Study Selection:**

Eligible studies included those with at least 10 probands with the defining feature of CH and/or severe cerebral ventriculomegaly that had undergone ES. Studies with fewer than 10 probands, studies of mild or moderate ventriculomegaly, and studies using genetic tests other than ES were excluded. A full-text review of 68 studies was conducted by 2 reviewers. Discrepancies were resolved by consensus.

**Data Extraction and Synthesis:**

Preferred Reporting Items for Systematic Reviews and Meta-Analyses guidelines and Meta-Analysis of Observational Studies in Epidemiology guidelines were used by 2 reviewers to extract data. Data were synthesized using a random-effects model of single proportions. Data analysis occurred in April 2023.

**Main Outcomes and Measures:**

The primary outcome was pooled diagnostic yield. Additional diagnostic yields were estimated for specific subgroups on the basis of clinical features, syndromic presentation, and parental consanguinity. For each outcome, a 95% CI and estimate of interstudy heterogeneity (*I^2^* statistic) was reported.

**Results:**

From 498 deduplicated and screened records, 9 studies with a total of 538 CH probands were selected for final inclusion. The overall diagnostic yield was 37.9% (95% CI, 20.0%-57.4%; *I^2^* = 90.1). The yield was lower for isolated and/or nonsyndromic cases (21.3%; 95% CI, 12.8%-31.0%; *I^2^* = 55.7). The yield was higher for probands with reported consanguinity (76.3%; 95% CI, 65.1%-86.1%; *I^2^* = 0) than those without (16.2%; 95% CI, 12.2%-20.5%; *I^2^* = 0).

**Conclusions and Relevance:**

In this systematic review and meta-analysis of the diagnostic yield of ES in CH, the diagnostic yield was concordant with that of previous recommendations for other neurodevelopmental disorders, suggesting that ES should also be recommended as a routine diagnostic adjunct for patients with CH.

## Key Points

**Question:**

What is the diagnostic yield of exome sequencing (ES) among patients with congenital hydrocephalus (CH), and does ES merit implementation as a first-tier diagnostic test in this population?

**Findings:**

This systematic review and meta-analysis included 538 probands with CH. The diagnostic yield of ES in CH was higher than that of the current recommendation for ES as a first-tier test for other neurodevelopmental disorders.

**Meaning:**

These findings suggest ES is a high-yield test for the molecular diagnosis of CH and should be recommended as such.

## Teaser

This systematic review and meta-analysis explores whether exome sequencing should be used as a test for the molecular diagnosis of congenital hydrocephalus.

## Introduction

Congenital hydrocephalus (CH) is a primary form of hydrocephalus characteristically marked by pathological expansion of the cerebral ventricles.^1^ CH is present in approximately 1 in 1000 live births and is among the most common neurodevelopmental disorders (NDDs) and structural brain disorders.^2^ In contrast with other NDDs, CH is often diagnosed postnatally or within the first year of life by radiological identification of cerebral ventriculomegaly and additional clinical and phenotypic features, such as macrocephaly. Prenatal methods depend largely on radiological identification of ventriculomegaly due to practical constraints of in utero diagnostics. Identification of severe ventriculomegaly is the principal (and often sole) diagnostic feature in prenatal CH cases.^3^

CH is a primary (idiopathic) disease and, by definition, lacks an identifiable clinical antecedent.^4,5^ Although clinical causes are unclear, the hallmark pathogenic cerebrospinal fluid accumulation can be associated with cerebral malformations such as aqueductal stenosis.^1,6^ Recent efforts to elucidate genetic factors have contributed to evidence of rare associated genetic variants in CH.^7,8^ Genetic factors are thought to contribute to both syndrome-associated and nonsyndromic (sporadic) CH^1^; however, although variants in more than 100 genes have been associated with syndromic forms of hydrocephalus, few have been associated with nonsyndromic forms.^9,10^ Despite efforts to elucidate genetic causes of nonsyndromic CH, the current body of associated variants accounts for only 5% of cases.^6^ It has been estimated that more than 40% of CH cases have genetic origins,^11^ and, thus, the vast majority of these cases remain to be elucidated.

Several studies have used exome sequencing (ES) in individuals with CH with varying results; some of these studies have identified associated variants in as many as 78% to 90% of cases.^12,13^ Due to the complex heterogeneity and implications of rare genetic variants in CH, using ES as a diagnostic tool might help uncover genetic factors associated with CH and aid in clinical management of patients.

Recently, 2 separate recommendations were released in support of ES as a first-line diagnostic test for individuals with NDDs. Srivastava et al^14^ used meta-analytic techniques to support ES as a high-yield diagnostic test for patients with global developmental delay (DD), intellectual disability (ID), and autism spectrum disorder. Subsequently, the American College of Medical Genetics and Genomics released clinical guidelines^15^ recommending ES for those with ID, DD, or congenital anomalies. Neither recommendation included CH as an NDD of interest. In this study, we focused on CH as a potential addition to these recommendations by testing the hypothesis that the diagnostic yield of ES in patients with CH is comparable to that of the previous guidelines^14,15^ establishing ES as a first-tier test for other NDDs.

## Methods

This systematic review and meta-analysis was conducted using the Preferred Reporting Items for Systematic Reviews and Meta-Analyses (PRISMA) reporting guideline.^16^ We also used the Meta-Analysis of Observational Studies in Epidemiology (MOOSE) reporting guideline.^17^

### Search Strategy and Information Sources

We searched PubMed, Cochrane Library, and Google Scholar to find relevant studies published in English using the following search terms: *congenital hydrocephalus*, *ventriculomegaly*, *cerebral ventriculomegaly*, *primary ventriculomegaly*, *fetal ventriculomegaly*, *prenatal ventriculomegaly*, *molecular analysis*, *genetic cause*, *genetic etiology*, *genetic testing*, *exome sequencing*, *whole exome sequencing*, *genome sequencing*, *microarray*, *microarray analysis*, and *copy number variants*. See eTable 1 in Supplement 1 for the combinations of these search terms. Due to the advent of ES in late 2009^18^ and early 2010,^19^ the search retrieved articles published between January 1, 2010, and the search date, April 10, 2023. Citations retrieved were screened using Covidence.^20^

### Eligibility Criteria and Selection Process

We included studies with CH or CH-like probands. The distinction between CH vs CH-like probands was determined by individual study author description. CH probands were explicitly described by the study authors as receiving a diagnosis of hydrocephalus. CH-like probands were fetal cases denoted only as receiving a diagnosis of severe cerebral ventriculomegaly, often precluded from a confirmed diagnosis of hydrocephalus due to prenatal constraints.^3^ Studies that only included cases of mild or moderate ventriculomegaly were not considered suggestive of CH^2,21^ and were excluded.

Studies eligible for inclusion included those with at least 10 probands with CH or severe ventriculomegaly who were undergoing ES. Exclusion criteria included studies performing ES with fewer than 10 probands with CH or ventriculomegaly, studies that did not discuss diagnostic yield, and studies not using ES (ie, using another genetic test such as chromosomal microarray or gene panel test).

To assess for inclusion criteria, search results were screened for relevance of titles and abstracts, and articles identified as relevant underwent full-text review. Following full-text review, articles meeting all eligibility criteria were selected for final inclusion (eTable 2 in Supplement 1).

### Risk of Bias Assessment

In compliance with current recommendations for meta-analyses of proportions with fewer than 10 studies,^22,23^ risk of bias was assessed qualitatively. We referenced the Risk of Bias in Nonrandomized Studies of Interventions tool.^24^

### Data Collection and Data Items

Data from included studies were populated into an extraction table by 2 independent reviewers (A.B.W.G. and N.H.M.). Data extracted included number of probands with positive ES (defined as pathogenic and likely pathogenic variants detected, for most articles) and the number of probands with negative ES (defined as variants of uncertain significance, likely benign, benign, or no variants detected, for most articles). Any discrepancies were resolved by consensus of the 2 reviewers. Grading of ventriculomegaly was determined by study authors and largely followed the convention of mild (10-12 mm), moderate (13-15 mm) and severe (≥16 mm).^3^

Secondary patient data were extracted for designation of patients into various subgroups for subsequent statistical analysis, including (1) clinical feature and diagnosis (CH or ventriculomegaly), (2) syndromic or nonsyndromic case, and (3) history of consanguinity. A proband’s clinical features were categorized as suggestive of syndromic CH according to (1) phenotype-based diagnosis of an associated syndrome and/or (2) implication of associative variation in a syndrome-associated gene. Phenotype-based diagnoses were determined by respective study authors, and syndrome-associated genes were denoted as such either by study author mention or by cross-reference with a list of known CH syndrome–associated genes.^9,13,25,26^ If an individual lacked either sign of syndromic CH, the patient was designated to the isolated, nonsyndromic group.

### Statistical Analysis

Using a random-effects model for meta-analyses of single proportions, the primary outcome (overall diagnostic yield) and subsequent comparisons of interest were evaluated. Freeman-Tukey double arcsine transformation was applied as the variance-stabilizing method for meta-analysis of single proportions,^27^ and a pooled diagnostic yield and 95% CI were calculated. As secondary comparisons, diagnostic yields were estimated for probands on the basis of (1) clinical feature (CH or ventriculomegaly); (2) isolated, nonsyndromic features; and (3) reported consanguinity in proband’s family. Interstudy heterogeneity was estimated by an *I^2^* statistic, with *P* < .05 denoting statistical significance. All analyses were conducted using SUMARI (JBI).^28^ Data analysis was conducted in April 2023.

## Results

### Study Selection

From the initial pool of 498 search results, 91 duplicate articles were removed before screening, and an additional 18 manually selected articles were added to the screening pool (eFigure in Supplement 1). At the title and abstract level, of the 425 articles screened, 357 were excluded. Of the 68 articles remaining for full-text review, 59 articles were excluded due to insufficient number of probands, use of genetic testing other than ES, lack of mention of molecular diagnostic yield, lack of specificity to CH, or overlap of cohort with another included study. At this stage, 10 additional articles^29,30,31,32,33,34,35,36,37,38^ were potentially eligible for inclusion but did not report data specific to CH or ventriculomegaly and/or ES yield. Corresponding authors of such articles were contacted via email by 1 of the reviewers (A.B.W.G.) with a request for supplemental data. Of the authors contacted, 1 provided supplemental data; however, the number of CH and ventriculomegaly probands was insufficient for inclusion, and the study^30^ was excluded. For the remaining 9 reports, none of the authors contacted provided supplemental data. Subsequently, 9 studies^12,13,26,39,40,41,42,43,44^ remained for final inclusion. One of the studies^40^ was a secondary analysis of 2 cohorts.^45,46^ Risk of bias was low for all included studies except for 1 domain grade of serious risk or no information for 1 study^39^ due to the nature of the report as a conference abstract (eTable 3 in Supplement 1).

### Study Characteristics

Individual study characteristics and demographics of the cohort of 538 probands from all 9 studies^12,13,26,39,40,41,42,43,44^ were tabulated as reported and as available in the original studies (Table). Overall, extracted cohorts included individuals with isolated and nonsyndromic CH, syndromic CH, and ventriculomegaly. Five studies^12,13,26,41,43^ included only CH probands, 1 study^42^ included both CH and ventriculomegaly probands, and 3 studies^39,40,44^ included only ventriculomegaly probands. All studies looking exclusively at cases with ventriculomegaly^39,40,44^ were fetal studies. All ventriculomegaly cases included had severe ventriculomegaly, except for probands from 1 included study,^39^ which only reported a combined, inextricable yield for moderate and severe ventriculomegaly cases.

**Table.** Characteristics of Included Studies

Study	Study population diagnosis	Type of sequencing	Total included probands, No.	Study location and cohort source	Study dates	Cohort inclusion criteria	Prenatal or postnatal cases	Isolated or syndromic cases	Consanguinity data	ACMG/AMP guidelines used
Alharbi et al, 2021^12^	CH	ES and singleton ES	10^a^	Tertiary care center in Saudi Arabia	April 2012-December 2018	Hydrocephalic patients with Walker-Warburg syndrome	Prenatal	Syndromic	Yes	Yes
Baptiste et al, 2022^40^	VM	Trio ES	18	PAGE and CUIMC	Not reported	Fetuses with anomalies of CNS^b^ and without isolated open NTD	Prenatal	Isolated	No	Yes
Jacquemin et al, 2023^41^	CH	Singleton ES^c^	28	Erasme Hospital, Belgium	Not reported	Primary CH probands (without *L1CAM* variation, abnormal karyotype, known syndromes, or NTD)	Both	Both	No (not patient-specific data)	Yes^d^
Jin et al, 2020^26^	CH	Trio ES trio and singleton ES	381	Yale University School of Medicine, USA	Not reported	Neurosurgically treated probands with primary CH	Postnatal	Both	Yes	No
Marangoni et al, 2021^42^	CH and VM	Trio ES;	10	Erasme Hospital, Belgium, and affiliated local hospitals	October 2016-June 2020	Fetuses with primary anomalies^b^ detected by ultrasound. Singleton cases were excluded	Prenatal	Both	Yes	Yes
Mei et al, 2021^43^	CH	Singleton ES	39^e^	Children’s Hospital of Fudan University, China	January 2016-December 2019	Infants with a diagnosis of hydrocephalus within first year of life	Postnatal	Both	No	No
Schindewolf et al, 2022^39^	VM	Singleton ES	14^f^	The Children’s Hospital of Philadelphia, USA	April 2016-August 2021	Patients referred for isolated VM; Mild isolated findings, such as mild VM, were excluded	Prenatal	Isolated	No	No
Shaheen et al, 2017^13^	CH	Singleton ES	27	King Faisal Specialist Hospital and Research Center, Saudi Arabia; Cincinnati Children’s Hospital, USA; and The Hospital for Sick Children in Toronto, Canada	Not reported	Families with at least 2 children diagnosed with CH	Both	Both	Yes	No
Yaron et al, 2022^44^	VM	Trio ES	11	Tel Aviv Sourasky Medical Center, Israel	2014 to 2021	Terminated pregnancies due to a major CNS ultrasound anomaly^g^ that were CMA-negative	Prenatal	Both	No	Yes

Abbreviations: ACMG, American College of Medical Genetics and Genomics; AMP, Association for Molecular Pathology; CH, congenital hydrocephalus; CMA, chromosomal microarray; CNS, central nervous system; CUIMC, Columbia University Irving Medical Center; ES, exome sequencing; NTD, neural tube defect; PAGE, Prenatal Assessment of Genomes and Exomes; VM, ventriculomegaly.

^a^ In this study, 1 of 11 total patients underwent gene panel testing and, thus, was excluded from meta-analysis.

^b^ Overall study cohort included patients without CH or severe VM. Only patients with features of CH or severe ventriculomegaly identified from the larger nonspecific cohort were included for meta-analysis.

^c^ Some additional siblings and parents (11) but not most siblings and parents also underwent ES.

^d^ In this study, ACMG guidelines were used to classify 5 of 8 positive cases as having a pathogenic or likely pathogenic variant, and the remaining 3 positive cases had associated deleterious variants in novel candidate CH genes graded outside of ACMG guidelines.

^e^ In this study, 40 of 110 probands had unclear clinical cause. Of the patients with unclear clinical cause, 1 was excluded from this meta-analysis because they underwent gene panel testing (not ES). The 70 of 110 probands with clear clinical cause (eg, intracranial hemorrhage or infection) were not included in this meta-analysis.

^f^ Cohort included inextricable yield of moderate and severe ventriculomegaly cases.

^g^ Overall study cohort included patients without CH or severe ventriculomegaly. Only patients with features of CH or severe ventriculomegaly identified from the larger nonspecific cohort were included for meta-analysis.

Eight studies^13,26,39,40,41,42,43,44^ included whole or partial cohorts with isolated and/or nonsyndromic cases allowing for targeted estimation of diagnostic yield. The remaining study^12^ with only syndromic CH individuals was excluded from the corresponding subcomparison. Four studies^12,13,26,42^ reported patient-level consanguinity data for the entire cohort, and the remaining 5 studies^39,40,41,43,44^ that did not report consanguinity were excluded from the subcomparison.

### Results of Syntheses

**Figure 1.**
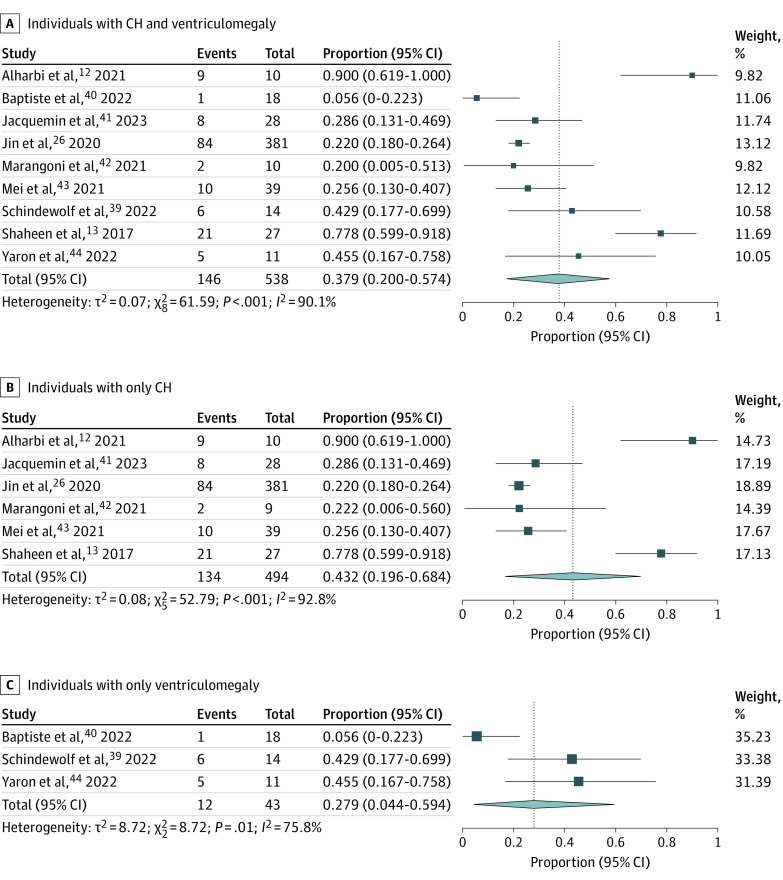
Diagnostic Yield of Exome Sequencing (ES) in All Patients Forest plots show the diagnostic yield of ES for patients with congenital hydrocephalus (CH) and ventriculomegaly (A), patients with only CH (B), and individuals with ventriculomegaly only (C). Events correspond to the number of individuals with associated variants identified by ES. The size of the square is proportional to the weight of the study in relation to the pooled estimate, and lines represent 95% CIs. The diamond represents the overall effect estimate of the meta-analysis.

To pool diagnostic yield from studies with disparate methods and/or populations, a random-effects meta-analysis was implemented. For the pooled cohort of 538 CH and ventriculomegaly probands from 9 studies,^12,13,26,39,40,41,42,43,44^ the random-effects methods revealed a diagnostic yield of 37.9% (95% CI, 20.0%-57.4%; *I^2^* = 90.1) (Figure 1A). For CH probands alone, the yield was higher (43.2%; 95% CI, 19.6%-68.4%; *I^2^* = 92.8) than the pooled CH and ventriculomegaly yield (37.9%) and higher than the yield of ventriculomegaly alone (27.9%; 95% CI, 4.4%-59.4%; *I^2^* = 75.8) (Figure 1B and Figure1C).

**Figure 2.**
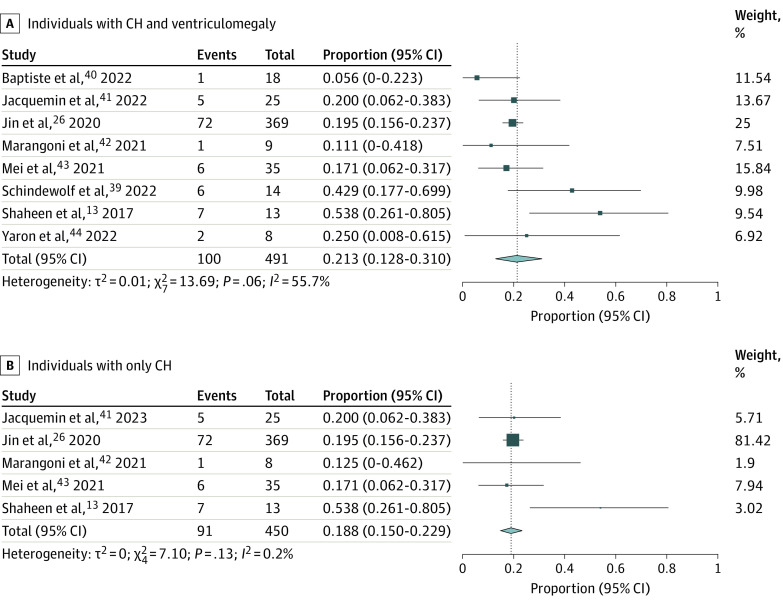
Diagnostic Yield of Exome Sequencing (ES) in Patients With Isolated and Nonsyndromic Cases Forest plots show the diagnostic yield of ES in patients with congenital hydrocephalus (CH) and ventriculomegaly (A) and patients with only CH (B). Events correspond to the number of individuals with associated variants identified by ES. The size of the square is proportional to the weight of the study in relation to the pooled estimate, and lines represent 95% CIs. The diamond represents the overall effect estimate of the meta-analysis.

**Figure 3.**
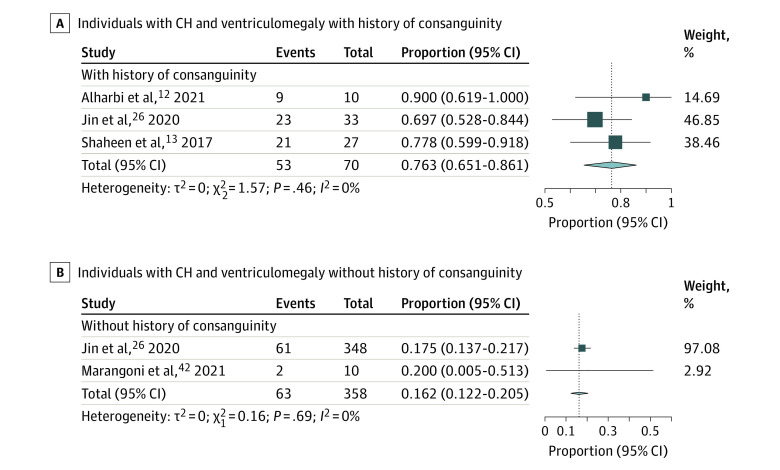
Diagnostic Yield of Exome Sequencing (ES) Among Patients With and Without History of Consanguinity Forest plots show the diagnostic yield of ES in patients with congenital hydrocephalus (CH) and ventriculomegaly among those who had a history of consanguinity (A) and those who did not (B). Events correspond to the number of individuals with associated variants identified by ES. The size of the square is proportional to the weight of the study in relation to the pooled estimate, and lines represent 95% CIs. The diamond represents the overall effect estimate of the meta-analysis.

For isolated and/or nonsyndromic cases, the yield for CH and ventriculomegaly probands was higher (21.3%; 95% CI, 12.8%-31.0%; *I^2^* = 55.7) (Figure 2A) than for CH probands alone (18.8%; 95% CI,15.0%-22.90%; *I^2^* = 0.2) (Figure 2B). For CH and ventriculomegaly probands with history of consanguinity, the yield was higher (76.3%; 95% CI, 65.1%-86.1%; *I^2^* = 0) (Figure 3A) than for those without reported consanguinity (16.2%; 95% CI, 12.2%-20.5%; *I^2^* = 0) (Figure 3B).

## Discussion

Our systematic review and meta-analysis of 9 studies^12,13,26,39,40,41,42,43,44^ combined 538 individuals with the defining feature of CH and/or primary ventriculomegaly. Compared with a recent meta-analysis^14^ heralding ES as a diagnostic test in patients with other NDDs (36%), the diagnostic yield from our CH-specific study (37.9%) was similar. Our calculated yield was higher for patients with only confirmed CH vs patients with only confirmed ventriculomegaly. For all patients with isolated and/or nonsyndromic cases, the yield was lower than for the pooled cohort. Furthermore, the yield was higher for those with a history of consanguinity than without. In sum, our results support expanding the recommendation of ES as a top-tier clinical test to CH diagnostics.

Despite becoming more accessible, ES still remains fairly cost-intensive and time-intensive.^47^ Thus, clinicians may lean toward implementing ES for cases that are more likely to harbor genetic factors, such as (1) confirmed hydrocephalic cases; (2) cases suggestive of isolated and/or nonsyndromic CH; and (3) cases with other factors associated with mendelian CH forms, such as history of consanguinity. Our results support implementation of ES in these cases with high mendelian risk.

Additionally, we argue that, as ES becomes more cost-efficient and time-efficient, ES should also be considered as a first-tier test for CH in all patients, including (1) unconfirmed prenatal cases suggestive of hydrocephalus, (2) cases with signs of syndromic associations, and (3) cases without risk factors such as consanguinity. Our evidence and reasoning are as follows.

First, for prenatal cases, detection of severe ventriculomegaly can be, but is not always, translated to a diagnosis of hydrocephalus.^3^ Implementing ES in prenatal CH-suggestive cases would allow for clearer delineation of benign and nonspecific vs pathogenic ultrasonographic findings. Furthermore, earlier CH diagnosis would allow for earlier postnatal treatment and, perhaps, better clinical outcomes.^48^ Allowing families and clinicians more time to provide tailored, informed care—emotionally, financially, clinically, and otherwise—for a newborn with a known CH diagnosis could increase quality of life for all involved.^4^ In our analysis, all ventriculomegaly cases were severe and prenatal. The diagnostic yield for CH cases was higher than for ventriculomegaly cases; however, the yield for ventriculomegaly alone (27.9%) is still considerable (when compared with the 36% yield in the previous guideline^14^ for ES in NDDs), and so we recommend that ES also be considered in prenatal cases with isolated, severe ventriculomegaly suggestive of CH.

Second, the question of ES for syndromic CH surrounds the necessity, not the efficacy, of this comprehensive test as opposed to a more targeted, less expensive, and faster option (eg, gene panel). For most syndromic cases, an associated variant could likely be detected by a gene panel of the more than 100 known syndrome-associated genes^9^; however, there is still value in ES for syndromic cases. Although genetic and clinical efforts to elucidate syndromic forms have been successful relative to nonsyndromic forms,^9,10^ proper detection and understanding of phenotypic presentation of syndromic forms can be nebulous. For example, some individuals with identified variants in known syndromic genes can clinically present as isolated CH cases.^41^ This phenomenon highlights the uncertainty in detecting CH syndromes. In addition to phenotypic uncertainty, CH syndromes can also present with genetic uncertainty and heterogeneity. One study^49^ noted that some patients with variants in the known CH-associated gene, *L1CAM*, had a negative prenatal targeted gene panel and later received a diagnosis by ES only. Offering ES for patients with symptoms suggestive of syndromic CH, even those with established associated variants in syndrome-associated genes, can result in identification of additional, potentially clinically informative, associated variants in nonsyndromic genes.^26^ Thus, ES for syndromic CH can provide a more comprehensive and informative snapshot than panels targeted for syndromic genes alone. Targeted diagnostic panels may currently be a more efficient method for strictly syndromic CH forms, but ES continues to be a competitive alternative due to the heterogeneity of syndrome-associated forms.

Third, although our analysis suggests that ES in patients with history of consanguinity offers a disproportionately higher yield (76.3%) than for patients without (16.2%), patients without history of consanguinity still have a considerable yield and should not be excluded from these precise diagnostic methods. Furthermore, risk factors may not always be reported or detected; therefore, the absence of reported risk factors should not necessarily serve as a deterrent against offering ES. Thus, due to the clinical and genetic heterogeneity of CH, the substantial diagnostic yields in all analyzed subgroups, and the increasing accessibility of ES, we urge clinicians to consider ES as the premier clinical diagnostic test for all CH patients.

According to recent practice guidelines,^15^ genetic testing might not be offered for patients with CH without comorbid NDD. Many patients with CH would have to wait to develop an additional NDD for which ES is recommended (eg, ID or DD) before receiving genetic testing. This current paradigm would result in delayed care for patients with CH. Because CH can be diagnosed earlier than ID or DD, testing all CH probands would allow for a timely genetic diagnosis with potential improvement in clinical outcomes. Beyond diagnostics, increasing rates of CH sequencing will accelerate identification of CH genes and pathomechanisms and allow for new translational discoveries such as the association of variants with clinically relevant variables like neurosurgical outcome.

### Limitations

This study has limitations. Although risk of bias was low in most domains for the included studies, one exception was the inclusion of a non–peer reviewed conference abstract^39^ with serious risk. However, because risk of bias was low in all other domains, and the abstract contained all necessary data for inclusion, we included this report.

This meta-analysis included cases with CH or CH-like features, namely ventriculomegaly. Included studies denoting only ventriculomegaly as a clinical feature looked exclusively at fetal cases. We included cases from these fetal ventriculomegaly studies as having CH-like features because severe ventriculomegaly is often the sole feature for prenatal diagnosis of CH.^3^ To limit nonspecific and benign cases, we included cases with severe ventriculomegaly and excluded cases denoted as mild, moderate, or ungraded and unspecified. We excluded fetal cases with mild or moderate ventriculomegaly because the majority (>90% of mild cases) of these have been shown to be associated with typical neurodevelopmental outcomes and are nonspecific to CH.^21^

The inclusion of ventriculomegaly cases in this CH meta-analysis raises certain concerns. Although we attempted to limit nonspecific and benign cases, including severe ventriculomegaly may have introduced some nonspecific cases into our study. However, the number of ventriculomegaly cases was a fraction of the total cohort (43 of 538 probands), and we ran additional analyses to examine CH and ventriculomegaly alone (Figure 1). Another consideration is that Schindewolf et al^39^ presented an inextricable group of moderate and severe ventriculomegaly cases. We included this group in our meta-analysis. Furthermore, Schindewolf and colleagues^39^ used a grading scale skewed toward severe ventriculomegaly (mild, 10-11 mm; moderate, 12-15 mm; or severe, ≥15 mm). However, given the high yield of that individual study cohort,^39^ (42.9%), the inclusion of potentially nonspecific moderate cases and skewing toward more severe ventriculomegaly grades did not hamper the diagnostic yield in comparison with the standard overall yield set by our meta-analysis (37.9%).

Our study is also limited by the designation of syndromic vs isolated and/or nonsyndromic cases. We used multiple data sources, including study author genotypic and phenotypic report and our own cross-reference of associated variants with a list of known syndrome-associated genes, to categorize cases. However, definitive distinction between the 2 CH forms is difficult, especially since additional syndromic symptoms may develop over time and may not present at the time of clinical assessment. This is an added consideration when grading prenatal cases, which can present as isolated but may develop syndromic symptoms postnatally.^41^ Our categorization of patients depended solely on data available at the time of clinical assessment and study publication and is thus limited.

Additionally, we identified a low number of studies and/or patients in certain subanalyses. For example, only 2 studies^26,42^ were included in the subanalysis of patients without consanguinity (Figure 3). Furthermore, 1 study^42^ had only 1 patient with ventriculomegaly (with negative ES), and thus was ineligible for the ventriculomegaly-specific subanalysis (Figure 1).

## Conclusions

Our findings underscore the high yield of ES in CH. Given that the percentage of patients receiving a molecular diagnosis by ES in CH is comparable to that of the current recommendation for other NDDs, we conclude that ES should also be recommended as a first-tier clinical diagnostic test for CH.

## Notes

Supplement 1. **eTable 1.** Search Terms **eTable 2**. List of Citations Located and Reason for Inclusion or Exclusion **eFigure.** Search Results Flow Graphic **eTable 3.** Risk of Bias Assessment

Supplement 2.
Data Sharing Statement
