# Supplementary material for: Molecular Diagnostic Yield of Exome Sequencing in Patients With Congenital Hydrocephalus: A Systematic Review and Meta-Analysis
Source: JAMA Netw Open. 2023 Nov 22;6(11):e2343384. doi: 10.1001/jamanetworkopen.2023.43384 (PMC10665979; doi:10.1001/jamanetworkopen.2023.43384)
Supplement: Supplement 1. — eTable 1. Search Terms eTable 2. List of Citations Located and Reason for Inclusion or Exclusion eFigure. Search Results Flow Graphic eTable 3. Risk of Bias Assessment [file jamanetwopen-e2343384-s001.pdf]

Supplemental Online Content

Greenberg ABW, Mehta NH, Allington G, Jin SC, Moreno-De-Luca A, Kahle KT. Molecular diagnostic yield of exome sequencing in patients with congenital hydrocephalus: a systematic review and meta-analysis. *JAMA Netw Open*. 2023;6(11):e2343384.
doi:10.1001/jamanetworkopen.2023.43384

eTable 1. Search Terms
eTable 2. List of Citations Located and Reason for Inclusion or Exclusion
eFigure. Search Results Flow Graphic
eTable 3. Risk of Bias Assessment

This supplemental material has been provided by the authors to give readers additional information about their work.

© 2023 Greenberg ABW et al. *JAMA Network Open*

eTable 1. Search Terms

	Terms	Filters applied
#1	Congenital hydrocephalus	
#2	Ventriculomegaly	
#3	Cerebral ventriculomegaly	
#4	Primary ventriculomegaly	
#5	Primary cerebral ventriculomegaly	
#6	Fetal ventriculomegaly	
#7	Prenatal ventriculomegaly	
#8	Molecular analysis	
#9	Genetic cause	
#10	Genetic etiology	
#11	Genetic testing	
#12	Whole exome sequencing	
#13	Exome sequencing	
#14	Whole genome sequencing	
#15	Genome sequencing	
#16	Microarray	
#17	Microarray analysis	
#18	Copy number variants	
**Combined Query**	(#1 OR #2 OR #3 OR #4 OR #5 OR #6 OR #7) AND (#8 OR #9 OR #10 OR #11 OR #12 OR #13 OR #14 OR #15 OR #16 OR #17 OR #18)	From January 1st, 2010 to April 10th, 2023; English language.

Terms adapted from search strategy employed in: Gonzalez-Mantilla PJ, Hu Y, Myers SM, et al. Diagnostic yield of exome sequencing in cerebral palsy and implications for genetic testing guidelines: a systematic review and meta-analysis. JAMA Pediatr. 2023;177(5):472-478. doi:10.1001/jamapediatrics.2023.0008

© 2023 Greenberg ABW et al. *JAMA Network Open*

eTable 2. List of Citations Located and Reason for Inclusion or Exclusion

Title	Citation	Included/Excluded	Reason (if excluded)
The Utility of Whole Exome Sequencing in Patients with Intellectual Disability and Developmental Delay as a First-Tier Diagnostic Testing Strategy	Richardson, E.(2020). *The Utility of Whole Exome Sequencing in Patients With Intellectual Disability and Developmental Delay as a First-Tier Diagnostic Testing Strategy.* (Master's thesis). Retrieved from https://scholarcommons.sc.edu/etd/5717	Excluded	Diagnostic yield not discussed/inferable
Impaired neurogenesis alters brain biomechanics in a neuroprogenitor-based genetic subtype of congenital hydrocephalus.	Duy PQ, Weise SC, Marini C, et al. Impaired neurogenesis alters brain biomechanics in a neuroprogenitor-based genetic subtype of congenital hydrocephalus. *Nat Neurosci*. 2022;25(4):458-473. doi:10.1038/s41593-022-01043-3	Excluded	Diagnostic yield not discussed/inferable
Prenatal phenotyping of fetal tubulinopathies: A multicenter retrospective case series	Brar BK, Thompson MG, Vora NL, et al. Prenatal phenotyping of fetal tubulinopathies: A multicenter retrospective case series. *Prenat Diagn*. 2022;42(13):1686-1693. doi:10.1002/pd.6269	Excluded	Diagnostic yield not discussed/inferable
Prenatal findings and associated survival rates in fetal ventriculomegaly: A prospective observational study	Ryan GA, Start AO, Cathcart B, et al. Prenatal findings and associated survival rates in fetal ventriculomegaly: A prospective observational study. *Int J Gynaecol Obstet*. 2022;159(3):891-897. doi:10.1002/ijgo.14206	Excluded	Diagnostic yield not discussed/inferable
Whole-exome sequencing in the evaluation of fetal structural anomalies: a prospective cohort study	Petrovski S, Aggarwal V, Giordano JL, et al. Whole-exome sequencing in the evaluation of fetal structural anomalies: a prospective cohort study. *Lancet*. 2019;393(10173):758-767. doi:10.1016/S0140-6736(18)32042-7	Excluded	Diagnostic yield not discussed/inferable
Exome Sequencing as a Potential Diagnostic Adjunct in Sporadic Congenital Hydrocephalus.	Sullivan W, Reeves BC, Duy PQ, et al. Exome Sequencing as a Potential Diagnostic Adjunct in Sporadic Congenital Hydrocephalus. *JAMA Pediatr*. 2021;175(3):310-313. doi:10.1001/jamapediatrics.2020.4878	Excluded	Not enough patients
An X-linked syndrome with severe neurodevelopmental delay, hydrocephalus, and early lethality caused by a missense variation in the OTUD5 gene	Tripolszki K, Sasaki E, Hotakainen R, et al. An X-linked syndrome with severe neurodevelopmental delay, hydrocephalus, and early lethality caused by a missense variation in the OTUD5 gene. *Clin Genet*. 2021;99(2):303-308. doi:10.1111/cge.13873	Excluded	Not enough patients
The impact of rapid exome sequencing on medical management of critically ill children	Freed AS, Clowes Candadai SV, Sikes MC, et al. The Impact of Rapid Exome Sequencing on Medical Management of Critically Ill Children. *J Pediatr*. 2020;226:202-212.e1. doi:10.1016/j.jpeds.2020.06.020	Excluded	Not enough patients
Surprisingly good outcome in antenatal diagnosis of severe hydrocephalus related to CCDC88C deficiency.	Wallis M, Baumer A, Smaili W, et al. Surprisingly good outcome in antenatal diagnosis of severe hydrocephalus related to CCDC88C deficiency. *Eur J Med Genet*. 2018;61(4):189-196. doi:10.1016/j.ejmg.2017.12.002	Excluded	Not enough patients

© 2023 Greenberg ABW et al. *JAMA Network Open*

Exome sequencing improves genetic diagnosis of structural fetal abnormalities revealed by ultrasound	Carss KJ, Hillman SC, Parthiban V, et al. Exome sequencing improves genetic diagnosis of structural fetal abnormalities revealed by ultrasound. *Hum Mol Genet*. 2014;23(12):3269-3277. doi:10.1093/hmg/ddu038	Excluded	Not enough patients
Prenatal diagnosis of diencephalic-mesencephalic junction dysplasia: Fetal magnetic resonance imaging phenotypes, genetic diagnoses, and outcomes	Lawrence AK, Whitehead MT, Kruszka P, et al. Prenatal diagnosis of diencephalic-mesencephalic junction dysplasia: Fetal magnetic resonance imaging phenotypes, genetic diagnoses, and outcomes. *Prenat Diagn*. 2021;41(6):778-790. doi:10.1002/pd.5909	Excluded	Not enough patients
Expanding the KIF4A-associated phenotype	Kalantari S, Carlston C, Alsaleh N, et al. Expanding the KIF4A-associated phenotype. *Am J Med Genet A*. 2021;185(12):3728-3739. doi:10.1002/ajmg.a.62443	Excluded	Not enough patients
Diagnostic yield of genome sequencing for prenatal diagnosis of fetal structural anomalies	Wang Y, Greenfeld E, Watkins N, et al. Diagnostic yield of genome sequencing for prenatal diagnosis of fetal structural anomalies. *Prenat Diagn*. 2022;42(7):822-830. doi:10.1002/pd.6108	Excluded	Not enough patients
Trio-whole-exome sequencing and preimplantation genetic diagnosis for unexplained recurrent fetal malformations	Guo W, Lai Y, Yan Z, et al. Trio-whole-exome sequencing and preimplantation genetic diagnosis for unexplained recurrent fetal malformations. *Hum Mutat*. 2020;41(2):432-448. doi:10.1002/humu.23935	Excluded	Not enough patients
Expansion of phenotype and genotypic data in CRB2-related syndrome	Lamont RE, Tan WH, Innes AM, et al. Expansion of phenotype and genotypic data in CRB2-related syndrome. *Eur J Hum Genet*. 2016;24(10):1436-1444. doi:10.1038/ejhg.2016.24	Excluded	Not enough patients
Molecular autopsy by trio exome sequencing (ES) and postmortem examination in fetuses and neonates with prenatally identified structural anomalies	Quinlan-Jones E, Lord J, Williams D, et al. Molecular autopsy by trio exome sequencing (ES) and postmortem examination in fetuses and neonates with prenatally identified structural anomalies. *Genet Med*. 2019;21(5):1065-1073. doi:10.1038/s41436-018-0298-8	Excluded	Not enough patients
Clinical Utility of Medical Exome Sequencing: Expanded Carrier Screening for Patients Seeking Assisted Reproductive Technology in China	Tong K, He W, He Y, et al. Clinical Utility of Medical Exome Sequencing: Expanded Carrier Screening for Patients Seeking Assisted Reproductive Technology in China. *Front Genet*. 2022;13:943058. Published 2022 Aug 22. doi:10.3389/fgene.2022.943058	Excluded	Not enough patients
Novel and recurrent variants identified in fetuses with central nervous system abnormalities by trios-medical exome sequencing	Tan H, Xie Y, Chen F, et al. Novel and recurrent variants identified in fetuses with central nervous system abnormalities by trios-medical exome sequencing. *Clin Chim Acta*. 2020;510:599-604. doi:10.1016/j.cca.2020.08.018	Excluded	Not enough patients

© 2023 Greenberg ABW et al. *JAMA Network Open*

Panventriculomegaly with a wide foramen of Magendie and large cisterna magna.	Kageyama H, Miyajima M, Ogino I, et al. Panventriculomegaly with a wide foramen of Magendie and large cisterna magna. *J Neurosurg*. 2016;124(6):1858-1866. doi:10.3171/2015.6.JNS15162	Excluded	Not enough patients
Acceleration and plateau: two patterns and outcomes of isolated severe fetal cerebral ventricular dilation	Ge CJ, Polan RM, Baranano KW, et al. Acceleration and plateau: two patterns and outcomes of isolated severe fetal cerebral ventricular dilation. *J Matern Fetal Neonatal Med*. 2021;34(18):3014-3020. doi:10.1080/14767058.2019.1677590	Excluded	Not enough patients
Bi-allelic loss-of-function variants in KIF21A cause severe fetal akinesia with arthrogryposis multiplex	Falb RJ, Müller AJ, Klein W, et al. Bi-allelic loss-of-function variants in *KIF21A* cause severe fetal akinesia with arthrogryposis multiplex. *J Med Genet*. 2023;60(1):48-56. doi:10.1136/jmedgenet-2021-108064	Excluded	Not enough patients
Retrospective analysis of a clinical exome sequencing cohort reveals the mutational spectrum and identifies candidate disease–associated loci for BAFopathies	Chen CA, Lattier J, Zhu W, et al. Retrospective analysis of a clinical exome sequencing cohort reveals the mutational spectrum and identifies candidate disease-associated loci for BAFopathies. *Genet Med*. 2022;24(2):364-373. doi:10.1016/j.gim.2021.09.017	Excluded	Not enough patients
PUF60 variants cause a syndrome of ID, short stature, microcephaly, coloboma, craniofacial, cardiac, renal and spinal features	Low KJ, Ansari M, Abou Jamra R, et al. PUF60 variants cause a syndrome of ID, short stature, microcephaly, coloboma, craniofacial, cardiac, renal and spinal features. *Eur J Hum Genet*. 2017;25(5):552-559. doi:10.1038/ejhg.2017.27	Excluded	Not enough patients
Trio-based low-pass genome sequencing reveals characteristics and significance of rare copy number variants in prenatal diagnosis	Chau MHK, Qian J, Chen Z, et al. Trio-Based Low-Pass Genome Sequencing Reveals Characteristics and Significance of Rare Copy Number Variants in Prenatal Diagnosis. *Front Genet*. 2021;12:742325. Published 2021 Sep 20. doi:10.3389/fgene.2021.742325	Excluded	Not enough patients
Disturbed Wnt Signalling due to a Mutation in CCDC88C Causes an Autosomal Recessive Non-Syndromic Hydrocephalus with Medial Diverticulum	Ekici AB, Hilfinger D, Jatzwauk M, et al. Disturbed Wnt Signalling due to a Mutation in CCDC88C Causes an Autosomal Recessive Non-Syndromic Hydrocephalus with Medial Diverticulum. *Mol Syndromol*. 2010;1(3):99-112. doi:10.1159/000319859	Excluded	Not enough patients
Abnormal Sylvian fissure at 20-30 weeks as indicator of malformations of cortical development: role of prenatal whole-genome sequencing	Liao Y, Yang Y, Wen H, Wang B, Zhang T, Li S. Abnormal Sylvian fissure at 20-30 weeks as indicator of malformations of cortical development: role of prenatal whole-genome sequencing. *Ultrasound Obstet Gynecol*. 2022;59(4):552-555. doi:10.1002/uog.24771	Excluded	Not enough patients
Clinical application of medical exome sequencing for prenatal diagnosis of fetal structural anomalies	Chen M, Chen J, Wang C, et al. Clinical application of medical exome sequencing for prenatal diagnosis of fetal structural anomalies. *Eur J Obstet*	Excluded	Not enough patients

© 2023 Greenberg ABW et al. *JAMA Network Open*

	Gynecol Reprod Biol. 2020;251:119-124. doi:10.1016/j.ejogrb.2020.04.033		
Genetic tests aid in counseling of fetuses with cerebellar vermis defects	Li L, Fu F, Li R, et al. Genetic tests aid in counseling of fetuses with cerebellar vermis defects. *Prenat Diagn*. 2020;40(10):1228-1238. doi:10.1002/pd.5732	Excluded	Not enough patients
Simultaneous Detection of CNVs and SNVs Improves the Diagnostic Yield of Fetuses with Ultrasound Anomalies and Normal Karyotypes	Qi Q, Jiang Y, Zhou X, et al. Simultaneous Detection of CNVs and SNVs Improves the Diagnostic Yield of Fetuses with Ultrasound Anomalies and Normal Karyotypes. *Genes (Basel)*. 2020;11(12):1397. Published 2020 Nov 25. doi:10.3390/genes11121397	Excluded	Not enough patients
Whole Genome Sequencing in the Evaluation of Fetal Structural Anomalies: A Parallel Test with Chromosomal Microarray Plus Whole Exome Sequencing	Zhou J, Yang Z, Sun J, et al. Whole Genome Sequencing in the Evaluation of Fetal Structural Anomalies: A Parallel Test with Chromosomal Microarray Plus Whole Exome Sequencing. *Genes (Basel)*. 2021;12(3):376. Published 2021 Mar 6. doi:10.3390/genes12030376	Excluded	Not enough patients
Fetal exome sequencing: yield and limitations in a tertiary referral center	Daum H, Meiner V, Elpeleg O, Harel T; collaborating authors. Fetal exome sequencing: yield and limitations in a tertiary referral center. *Ultrasound Obstet Gynecol*. 2019;53(1):80-86. doi:10.1002/uog.19168	Excluded	Not enough patients
Whole-exome sequencing increases the diagnostic rate for prenatal fetal structural anomalies	Lei L, Zhou L, Xiong JJ. Whole-exome sequencing increases the diagnostic rate for prenatal fetal structural anomalies. *Eur J Med Genet*. 2021;64(9):104288. doi:10.1016/j.ejmg.2021.104288	Excluded	Not enough patients
X-Linked Hydrocephalus with New L1CAM Pathogenic Variants: Review of the Most Prevalent Molecular and Phenotypic Features	Ahmed RR, Medhat AM, Hamdy GM, Effat LKE, Abdel-Hamid MS, Abdel-Salam GMH. X-Linked Hydrocephalus with New L1CAM Pathogenic Variants: Review of the Most Prevalent Molecular and Phenotypic Features. *Molecular Syndromology* 2023;():1-10.	Excluded	Not ES
Prenatal molecular diagnosis of a severe type of L1 syndrome (X-linked hydrocephalus)	Yamasaki M, Nonaka M, Suzumori N, et al. Prenatal molecular diagnosis of a severe type of L1 syndrome (X-linked hydrocephalus). *J Neurosurg Pediatr*. 2011;8(4):411-416. doi:10.3171/2011.7.PEDS10531	Excluded	Not ES
Congenital brain malformations in Sudanese children: an outpatient-based study	Mohammed IN, Suliman SA, Elseed MA, Hamed AA, Babiker MO, Taha SO. Congenital brain malformations in Sudanese children: an outpatient-based study. *Sudan J Paediatr*. 2018;18(1):48-56. doi:10.24911/SJP.2018.1.7	Excluded	Not ES
Congenital hydrocephalus in clinical practice: a genetic diagnostic approach.	Verhagen JM, Schrander-Stumpel CT, Krapels IP, et al. Congenital hydrocephalus in clinical practice: a genetic diagnostic approach. *Eur J Med Genet*. 2011;54(6):e542-e547. doi:10.1016/j.ejmg.2011.06.005	Excluded	Not ES

Prenatal Neurologic Diagnosis: Challenges in Neuroimaging, Prognostic Counseling, and Prediction of Neurodevelopmental Outcomes	Agarwal S, Tarui T, Patel V, Turner A, Nagaraj U, Venkatesan C. Prenatal Neurological Diagnosis: Challenges in Neuroimaging, Prognostic Counseling, and Prediction of Neurodevelopmental Outcomes. *Pediatr Neurol*. 2023;142:60-67. doi:10.1016/j.pediatrneurol.2023.02.013	Excluded	Not ES
Impaired methylation modifications of FZD3 alter chromatin accessibility and are involved in congenital hydrocephalus pathogenesis	Wang L, Shangguan S, Chang S, et al. Impaired methylation modifications of FZD3 alter chromatin accessibility and are involved in congenital hydrocephalus pathogenesis. *Brain Res*. 2014;1569:48-56. doi:10.1016/j.brainres.2014.04.010	Excluded	Not ES
Novel missense mutation of L1CAM in a fetus with isolated hydrocephalus	Duan H, Zhao G, Wang Y, Zhu X, Li J. Novel missense mutation of L1CAM in a fetus with isolated hydrocephalus. *Congenit Anom (Kyoto)*. 2018;58(5):176-177. doi:10.1111/cga.12267	Excluded	Not ES
Adducted thumbs: a clinical clue to genetic diagnosis.	Verhagen JM, Schrander-Stumpel CT, Blezer MM, et al. Adducted thumbs: a clinical clue to genetic diagnosis. *Eur J Med Genet*. 2013;56(3):153-158. doi:10.1016/j.ejmg.2012.11.004	Excluded	Not ES
Accuracy of diagnosis and counseling of fetal brain anomalies prior to 24 weeks of gestational age	Snoek R, Albers MEWA, Mulder EJH, et al. Accuracy of diagnosis and counseling of fetal brain anomalies prior to 24 weeks of gestational age. *J Matern Fetal Neonatal Med*. 2018;31(16):2188-2194. doi:10.1080/14767058.2017.1338258	Excluded	Not ES
Impact of introduction of noninvasive prenatal testing on uptake of genetic testing in fetuses with central nervous system anomalies	Al Toukhi S, Chitayat D, Keunen J, et al. Impact of introduction of noninvasive prenatal testing on uptake of genetic testing in fetuses with central nervous system anomalies. *Prenat Diagn*. 2019;39(7):544-548. doi:10.1002/pd.5466	Excluded	Not ES
Spectrum and Detection Rate of L1CAM Mutations in Isolates and Familial Cases with Clinically Suspected L1-Disease	Finckh U, Schröder J, Ressler B, Veske A, Gal A. Spectrum and detection rate of L1CAM mutations in isolated and familial cases with clinically suspected L1-disease. *Am J Med Genet*. 2000;92(1):40-46. doi:10.1002/(sici)1096-8628(20000501)92:1<40::aid-ajmg7>3.0.co;2-r	Excluded	Not ES
Genotype-first in a cohort of 95 fetuses with multiple congenital abnormalities: when exome sequencing reveals unexpected fetal phenotype-genotype correlations	Lefebvre M, Bruel AL, Tisserant E, et al. Genotype-first in a cohort of 95 fetuses with multiple congenital abnormalities: when exome sequencing reveals unexpected fetal phenotype-genotype correlations. *J Med Genet*. 2021;58(6):400-413. doi:10.1136/jmedgenet-2020-106867	Excluded	Not specific to CH
Whole-exome sequencing on deceased fetuses with ultrasound anomalies: expanding our knowledge of	Yates CL, Monaghan KG, Copenheaver D, et al. Whole-exome sequencing on deceased fetuses with ultrasound anomalies: expanding our knowledge of	Excluded	Not specific to CH

© 2023 Greenberg ABW et al. *JAMA Network Open*

genetic disease during fetal development	genetic disease during fetal development. *Genet Med*. 2017;19(10):1171-1178. doi:10.1038/gim.2017.31		
Genome sequencing combining prenatal ultrasound in the evaluation of fetal CNS structural anomalies	Yang Y, Zhao S, Sun G, Chen F, Zhang T, Song J, Yang W, Wang L, Zhan N, Yang X. Genome sequencing combining prenatal ultrasound in the evaluation of fetal CNS structural anomalies. *medRxiv* 2020;():2020-03.	Excluded	Not specific to CH
Mutations of ADAMTS9 Cause Nephronophthisis-Related Ciliopathy.	Choi YJ, Halbritter J, Braun DA, et al. Mutations of ADAMTS9 Cause Nephronophthisis-Related Ciliopathy. *Am J Hum Genet*. 2019;104(1):45-54. doi:10.1016/j.ajhg.2018.11.003	Excluded	Not specific to CH
Clinical application of whole-exome sequencing across clinical indications	Retterer K, Juusola J, Cho MT, et al. Clinical application of whole-exome sequencing across clinical indications. *Genet Med*. 2016;18(7):696-704. doi:10.1038/gim.2015.148	Excluded	Not specific to CH
Molecular autopsy by proxy in preconception counseling	Ali Alghamdi M, Alrasheedi A, Alghamdi E, et al. Molecular autopsy by proxy in preconception counseling. *Clin Genet*. 2021;100(6):678-691. doi:10.1111/cge.14049	Excluded	Not specific to CH
A Retrospective Analysis of Clinically Focused Exome Sequencing Results of 372 Infants with Suspected Monogenic Disorders in China	Jia A, Lei Y, Liu DP, Pan L, Guan HZ, Yang B. A Retrospective Analysis of Clinically Focused Exome Sequencing Results of 372 Infants with Suspected Monogenic Disorders in China. *Pharmgenomics Pers Med*. 2023;16:81-97. Published 2023 Feb 2. doi:10.2147/PGPM.S387767	Excluded	Not specific to CH
Diagnostic power and clinical impact of exome sequencing in a cohort of 500 patients with rare diseases	Quaio CRDC, Moreira CM, Novo-Filho GM, et al. Diagnostic power and clinical impact of exome sequencing in a cohort of 500 patients with rare diseases. *Am J Med Genet C Semin Med Genet*. 2020;184(4):955-964. doi:10.1002/ajmg.c.31860	Excluded	Not specific to CH
Genome sequencing combining prenatal ultrasound in the evaluation of fetal CNS structural abnormalities	Yang Y, Zhao S, Sun G, et al. Genome sequencing combining prenatal ultrasound in the evaluation of fetal CNS structural anomalies. Preprint. medRxiv. Posted online March 06, 2020. doi:10.1101/2020.03.04.20031294	Excluded	Not specific to CH
Molecular Findings Among Patients Referred for Clinical Whole-Exome Sequencing	Yang Y, Muzny DM, Xia F, et al. Molecular findings among patients referred for clinical whole-exome sequencing. *JAMA*. 2014;312(18):1870-1879. doi:10.1001/jama.2014.14601	Excluded	Not specific to CH
The genetic background of hydrocephalus in a population-based cohort: implication of ciliary movement	Munch TN, Hedley PL, Hagen CM, et al. The genetic background of hydrocephalus in a population-based cohort: implication of ciliary involvement. *Brain Commun*. 2023;5(1):fcad004. Published 2023 Jan 10. doi:10.1093/braincomms/fcad004	Excluded	Not specific to CH

© 2023 Greenberg ABW et al. *JAMA Network Open*

Whole exome sequencing as a diagnostic adjunct to clinical testing in fetuses with structural abnormalities	Fu F, Li R, Li Y, et al. Whole exome sequencing as a diagnostic adjunct to clinical testing in fetuses with structural abnormalities. *Ultrasound Obstet Gynecol*. 2018;51(4):493-502. doi:10.1002/uog.18915	Excluded	Not specific to CH
Prenatal exome sequencing in 65 fetuses with abnormality of the corpus callosum: contribution to further diagnostic delineation	Heide S, Spentchian M, Valence S, et al. Prenatal exome sequencing in 65 fetuses with abnormality of the corpus callosum: contribution to further diagnostic delineation. *Genet Med*. 2020;22(11):1887-1891. doi:10.1038/s41436-020-0872-8	Excluded	Not specific to CH
GemC1 is a critical switch for neural stem cell generation in the postnatal brain	Lalioti ME, Kaplani K, Lokka G, et al. GemC1 is a critical switch for neural stem cell generation in the postnatal brain. *Glia*. 2019;67(12):2360-2373. doi:10.1002/glia.23690	Excluded	Overlapping cohort
De Novo Mutation in Genes Regulating Neural Stem Cell Fate in Human Congenital Hydrocephalus.	Furey CG, Choi J, Jin SC, et al. De Novo Mutation in Genes Regulating Neural Stem Cell Fate in Human Congenital Hydrocephalus. *Neuron*. 2018;99(2):302-314.e4. doi:10.1016/j.neuron.2018.06.019	Excluded	Overlapping cohort
Prenatal exome sequencing analysis in fetal structural anomalies detected by ultrasonography (PAGE): a cohort study	Lord J, McMullan DJ, Eberhardt RY, et al. Prenatal exome sequencing analysis in fetal structural anomalies detected by ultrasonography (PAGE): a cohort study. *Lancet*. 2019;393(10173):747-757. doi:10.1016/S0140-6736(18)31940-8	Excluded	Overlapping cohort
Neuroimaging manifestations and genetic heterogeneity of Walker-Warburg syndrome in Saudi patients	Alharbi S, Alhashem A, Alkuraya F, Kashlan F, Tlili-Graiess K. Neuroimaging manifestations and genetic heterogeneity of Walker-Warburg syndrome in Saudi patients. Brain Dev. 2021;43(3):380-388. doi:10.1016/j.braindev.2020.10.012	Included	N/A
The genetic landscape of familial congenital hydrocephalus	Shaheen R, Sebai MA, Patel N, et al. The genetic landscape of familial congenital hydrocephalus. Ann Neurol. 2017;81(6):890-897. doi:10.1002/ana.24964	Included	N/A
Genetic etiology of prenatally detected isolated moderate to severe ventriculomegaly	Schindewolf E, DiCicco R, Miller K, et al. OP052: Genetic etiology of prenatally detected isolated moderate to severe ventriculomegaly. Genetics in Medicine. 2022;24(3):S377-S378. doi:10.1016/j.gim.2022.01.598	Included	N/A
Fetal central nervous system anomalies: When should we offer exome sequencing?	Baptiste C, Mellis R, Aggarwal V, et al. Fetal central nervous system anomalies: When should we offer exome sequencing?. Prenat Diagn. 2022;42(6):736-743. doi:10.1002/pd.6145	Included	N/A
Congenital hydrocephalus: new Mendelian mutations and evidence for oligogenic inheritance	Jacquemin V, Versbraegen N, Duerinckx S, et al. Congenital hydrocephalus: new Mendelian mutations and evidence for oligogenic inheritance. Hum Genomics.	Included	N/A

© 2023 Greenberg ABW et al. *JAMA Network Open*

	2023;17(1):16. Published 2023 Mar 2. doi:10.1186/s40246-023-00464-w		
Exome sequencing implicates genetic disruption of prenatal neuro-gliogenesis in sporadic congenital hydrocephalus	Jin SC, Dong W, Kundishora AJ, et al. Exome sequencing implicates genetic disruption of prenatal neuro-gliogenesis in sporadic congenital hydrocephalus. Nat Med. 2020;26(11):1754-1765. doi:10.1038/s41591-020-1090-2	Included	N/A
Implementation of fetal clinical exome sequencing: Comparing prospective and retrospective cohorts	Marangoni M, Smits G, Ceysens G, et al. Implementation of fetal clinical exome sequencing: Comparing prospective and retrospective cohorts. Genet Med. 2022;24(2):344-363. doi:10.1016/j.gim.2021.09.016	Included	N/A
Genetic etiologies associated with infantile hydrocephalus in a Chinese infantile cohort	Mei HF, Dong XR, Chen HY, et al. Genetic etiologies associated with infantile hydrocephalus in a Chinese infantile cohort. World J Pediatr. 2021;17(3):305-316. doi:10.1007/s12519-021-00429-w	Included	N/A
Exome sequencing as first-tier test for fetuses with severe central nervous system structural anomalies	Yaron Y, Ofen Glassner V, Mory A, et al. Exome sequencing as first-tier test for fetuses with severe central nervous system structural anomalies. Ultrasound Obstet Gynecol. 2022;60(1):59-67. doi:10.1002/uog.24885	Included	N/A

© 2023 Greenberg ABW et al. *JAMA Network Open*

eFigure. Search Results Flow Graphic (adapted from PRISMA 2020 Flow Diagram)[16]

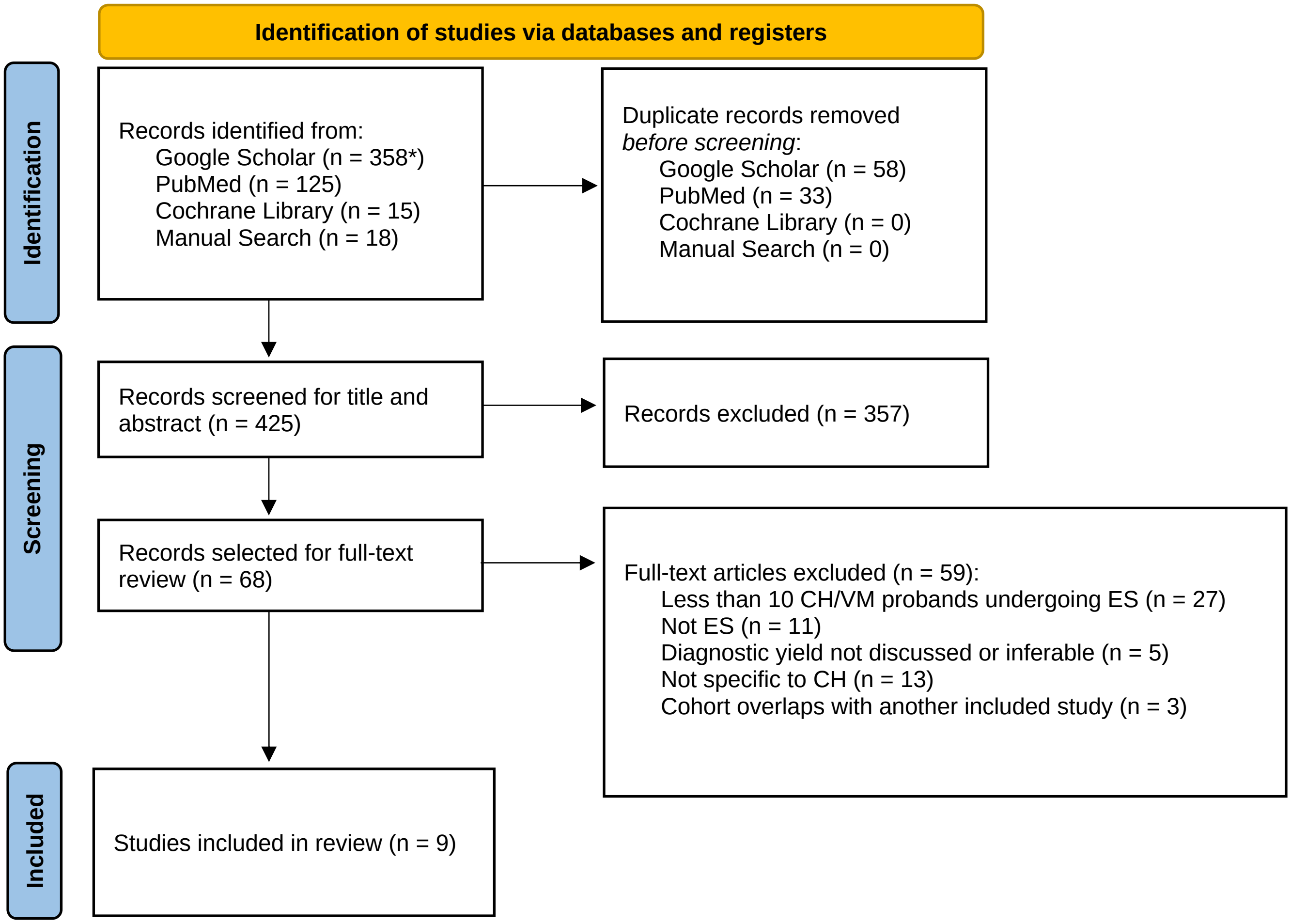

*Screened the first 300 (excluding 58 duplicate records) records of 1,200 records identified.

Abbreviations: CH = congenital hydrocephalus; VM = ventriculomegaly; ES = exome sequencing.

© 2023 Greenberg ABW et al. *JAMA Network Open*

eTable 3. Risk of Bias Assessment (adapted from ROBINS-I)[24]

**Study**	**Bias due to randomization**	**Bias due to missing data**	**Bias due to outcome measurement**	**Bias due to selection of reported result**
Alharbi et al., 2021	Low	Low	Low	Low
Baptiste et al., 2022	Low	Low	Low	Low
Jacquemin et al., 2023	Low	Low	Low	Low
Jin et al., 2020	Low	Low	Low	Low
Marangoni et al., 2021	Low	Low	Low	Low
Mei et al., 2021	Low	Low	Low	Low
Schindewolf et al., 2022	Low	Low	**Serious/No Information**	Low
Shaheen et al., 2017	Low	Low	Low	Low
Yaron et al., 2022	Low	Low	Low	Low

© 2023 Greenberg ABW et al. *JAMA Network Open*
