# Supplementary material for: Molecular Diagnostic Yield of Exome Sequencing in Patients With Congenital Hydrocephalus: A Systematic Review and Meta-Analysis
Source: JAMA Netw Open. 2023 Nov 22;6(11):e2343384. doi: 10.1001/jamanetworkopen.2023.43384 (PMC10665979; doi:10.1001/jamanetworkopen.2023.43384)
Supplement: Supplement 2. — Data Sharing Statement [file jamanetwopen-e2343384-s002.pdf]

Data Sharing Statement

Greenberg. Molecular Diagnostic Yield of Exome Sequencing in Patients With Congenital Hydrocephalus. *JAMA Netw Open*. Published November 22, 2023. doi:10.1001/jamanetworkopen.2023.43384

Data
Data available: No

Additional Information
Explanation for why data not available: All quantitative data used for this study have been included in the main text. Additional data may be available upon request from the corresponding author.
